# P083: Prevalence of multi-drug resistant tuberculosis and associated risk factors in HIV-positive patients registered at Mpilo Opportunistic Infection clinic, Bulawayo, Zimbabwe

**DOI:** 10.1186/2047-2994-2-S1-P83

**Published:** 2013-06-20

**Authors:** B Murwira Neemanyame

**Affiliations:** 1 National Tuberculosis Reference laboratory,Zimbabwe, Bulawayo, Zimbabwe

## Introduction

Tuberculosis (TB) is a major public health disease, affecting one third of the world’s population and killing approximately two million people yearly. The emergence of resistance to anti-tuberculosis drugs, particularly MDR-TB.

## Objectives

To determine prevalence of multidrug-resistant tuberculosis (MDR-TB) and associated risk factors among adult (≥18 years) HIV positive patients registered at Mpilo Opportunistic Infection (OI) clinic. To assess the association of CD4 count and MDR-TB.

## Methods

A health facility based cross-sectional study was carried out at Mpilo OI Clinic between 01 March and 31 July 2012. Convenience sampling was used to recruit 275 adult HIV positive patients into the study on a daily basis. A single sample for MDR-TB was collected from each one of these participants. A total of 275 sputum and aspirate(Bone marrow, Aspirates, pus Cerebrospinal fluid) samples were collected and cultured for MDR-TB using both the Liquid using BACTEC Mycobacterium Growth Indicator Tube 960 (MGIT) and the Conventional Solid Lowenstein Jensen (LJ) culture methods. Whole blood for CD4 count was collected from each participant and tested using BD FACS Calibur Flow Cytometry CD4 count machine. Logistic regression was used to determine predictors of MDR-TB prevalence.

## Results

The prevalence of MDR-TB was 2.6% among adult HIV patients registered at Mpilo OI Clinic and attended the clinic between 01 March and 31July 2012.In the multivariate analysis, MDR-TB prevalence was associated with CD4 count (OR 0.14 p=0.043).

## Conclusion

A prevalence of 2.6% of MDR-TB among HIV positive patients was found. This is very high considering this high MDR-TB risk group. A CD4 count of >200 cells/ul was found to be protective of high MDR-TB prevalence. Targeted interventions of MDR-TB are necessary to reduce incident MDR-TB cases among HIV positive patients. Increased MDR-TB case finding through culture and Drug Susceptibility testing before initiation of First line drugs is necessary to reduce mistreatment. Infection control measures need to be put in place to reduce transmission of MDR-TB.

## Disclosure of interest

None declared.

